# Novel Superhydrophobic Sand and Polyurethane Sponge Coated with Silica/Modified Asphaltene Nanoparticles for Rapid Oil Spill Cleanup

**DOI:** 10.3390/nano9020187

**Published:** 2019-02-02

**Authors:** Ayman M. Atta, Mahmood M. S. Abdullah, Hamad A. Al-Lohedan, Nermen H. Mohamed

**Affiliations:** 1 Chemistry Department, College of Science, King Saud University, Riyadh 11451, Saudi Arabia; maltaiar@ksu.edu.sa (M.M.S.A.); hlohedan@ksu.edu.sa (H.A.A.-L.); 2 Egyptian petroleum research institute, Nasr city, Cairo 11727, Egypt; nermenhefiny@yahoo.com

**Keywords:** hydrophobic silica nanoparticles, oil spill, superhydrophobic, wetting characteristics, superhydrophobic sand and sponge

## Abstract

Superhydrophobic nanomaterials are promising in the important pursuit to alleviate the environmental pollution caused by the petroleum crude oil industry, especially to clean-up oil spills. In this work, asphaltenes isolated from crude oil were modified to act as capping agents during the synthesis of hydrophobic silica nanoparticles (HSNPs). The chemical structure, surface morphology, particle size, and surfaces charge of HSNPs were investigated. The contact angles of water droplets on HSNP film surfaces were measured to investigate their wetting properties. Finally, superhydrophobic sand and polyurethane sponge were prepared by coating them with HSNPs and applied in the cleanup of oil spills of viscous heavy Arabian crude oil.

## 1. Introduction

Petroleum crude oil spilled into aquatic environments during their off-shore well production, disasters, crude oil production, transportation, and refining of the petroleum products has disastrous kinematic viscosity of 88 cSt at 37.7 °C ecosystems. Over billions of dollars are spent in the control of pollution due to petroleum crude oil spills via different methods and techniques to clean up the mess and avoid the loss of valuable energy resources [1]. Many methods based on mechanical, physical, chemical, and bioremediation are employed to alleviate and control the pollution occurring from the oil spills [1,2,3]. Chemicals such as dispersants, emulsifiers, or demulsifiers, and oil absorbers are widely used, among various other expensive techniques [4,5]. The applications of surfactants as dispersants, emulsifiers, and demulsifiers were effective techniques for treating the oil spill dispersed in aquatic environments without recovery of the crude oil is not an economically feasible method. These limitations provide motivation to develop new oil spill recovery technologies. Nowadays, the fabrication of new inexpensive materials or devices with excellent efficiency to recover the oil floating on the water surfaces at a rapid rate with selective recovery of oil over water without energy input, and the durability and reusability of such materials are the main targets for the production of valuable oil collectors. The dispersion of hydrophobic magnetic fluids in the oil spill followed by the collection of the mixture with an external magnetic field was developed to control the oil spill pollution [6,7,8,9]. In addition, hydrophobic meshes were successfully developed as oil recovery devices to continuously separate oil from water to recover the crude oil spill from the water surface [10,11,12]. Consequently, fabrication of superhydrophobic materials (with a water contact angle > 150°) is proposed as the cost-effective plan to clean up the oil pollutants from the water surface [13,14,15]. The challenges provide motivation for the development of new oil spill recovering technologies based on the production of superhydrophobic nanomaterials to form a rough surface coating on dust, sand, fibers, foams, and sponges with controlled wettability [16,17,18].

The fabrications of superhydrophobic coatings using electrospinning or airbrushing polymers [19,20], carbon nanotubes, [21,22], chemical etching [23,24], assembled long chain fatty acids [25], fluorinated growing zinc oxide crystals, and perfluorinated polyethylene glycol surfactants [26,27] have been reported. Spray coating of hydrophobic nanoparticles/polymer suspensions on the surfaces of different substrates such as sand, dust, fibers, and steel meshes has been used to produce superhydrophobic surfaces [28,29,30]. The objective of this work is to modify the chemical structure of solid petroleum products of the crude oil, such as waxes, asphaltenes, and petroleum coke, and use them as hydrophobic and inexpensive materials to be applied as oil spill cleanup materials. Previously, hydrophobic sand coated with paraffin waxes was used directly to separate oil from water [31]. The hydrophobic sand could be dispersed in the crude oil to increase the specific gravity of crude oil to be easily separated using skimmers without the formation of emulsions. In our previous work [7], the chemical structure of asphaltenes was modified to apply them as hydrophobic magnetic oil spill collectors. In this work, superhydrophobic silica nanoparticles capped with chemically modified asphaltenes were produced and coated on sand or polyurethane foam to obtain materials with superior recyclability, good mechanical strength, low cost, and manufacture scalability. Toward this, hydrophobic silane precursors were prepared to interact with modified asphaltene-maleic anhydride adducts to form superhydrophobic silica after alkaline hydrolysis with tetraethoxysilane to generate superhydrophobic surfaces. The reactive groups of superhydrophobic silica interacted with sand and/or polyurethane sponge to render them more hydrophobic. The superhydrophobicity of the modified sand or polyurethane sponge and their efficiencies as oil spill collectors were investigated. The results provide a basis for the rational design and implementation of such materials in the oil spill recovery with good recyclability.

## 2. Materials and Methods 

### 2.1. Materials

All materials used in the present work were of analytical grade and purchased from Sigma–Aldrich chemicals Co. (St. Louis, MO, USA). Silicone precursors based on tetraethoxysilane (TEOS), vinyl trimethoxysilane (VTS), and γ-aminopropyltriethoxysilane (APS), and oleylamine (OA) were used to prepare hydrophobic silicone precursors. Polydimethylsiloxane (PDMS prepolymer; Sylgard 184A) and a thermal curing agent (Sylgard 184B) were used to modify the surface of polyurethane sponge. Commercial polyurethane sponge (apparent density, 0.032 g/cm^3^; void volume, >97%) was purchased from a local furniture store (Riyadh, Saudi Arabia). Toluene: *n*-heptane (1:40 vol.%) mixture was used to isolate the asphaltene fractions from crude oil. Arabian heavy crude oil (API gravity of 19.2°, kinematic viscosity of 88 cSt at 37.7 °C; asphaltenes, wax, aromatics, resin, and saturates contents of 15, 3, 52, 14, and 16 wt.%, respectively, kinematic viscosity of 48 cSt at 16 °C; asphaltenes, wax, aromatics, resin, and saturates contents of 6, 8, 43, 18, and 23 wt.%, respectively) and medium crude oil (27.4° API; 48 cSt kinematic viscosity at 16 °C; asphaltenes, wax, aromatics, resin and saturates contents were 10, 6, 43, 18, and 23 wt.%, respectively) were produced by Aramco, Dammam, Saudi Arabia. Seawater was collected from the Arabian Gulf (Al-Khobar, Saudi Arabia). 

### 2.2. Preparation Methods

(a) Preparation of asphaltene succinimide derivative of γ-aminopropyltriethoxysilane (ASAS)

Asphaltene (2 g) and maleic anhydride (1.5 g) were dissolved in 50 mL of toluene in a three-necked round bottom flask. The mixture was refluxed for 8 h under nitrogen atmosphere, followed by the removal of solvent using a rotary evaporator under reduced pressure. The product of this reaction was mixed with an equimolar amount of APS and 5 mL of toluene and the mixture was refluxed at 165 °C for 2.5 h. The reaction temperature increased up to 180 °C for 4 h. Finally, the mixture was cooled to room temperature and washed thrice with acetone followed by water. The asphaltene succinimide derivative of γ-aminopropyltriethoxysilane (ASAS) was obtained at a yield of 85.7% at the end.

(b) Preparation of amino oleyl ethylltrimethoxysilane (AOS)

OA (0.01 mol) was added to VTS (0.05 mol) in 80 mL tetrahydrofuran (THF) with stirring at 65 °C for 4 h. Then, THF was removed using a rotary evaporator under reduced pressure to obtain amino oleyl ethylltrimethoxysilane (AOS). The Si content (6.75 wt.%) determined via inductively coupled plasma atomic emission spectrometry (ICP-AES) confirms the formation of AOS. The nitrogen content (3.33 wt.%) and reaction yield (92%) also confirm the formation of an adduct of OA and VTS to form AOS.

(c) Preparation of hydrophobic silica nanoparticles 

Hydrophobic silica nanoparticles, HSNP-1, were prepared by the hydrolysis of silicon precursors in methanol in the presence of an ammonia solution. For this, methanol (100 mL) was sonicated for 10 min with TEOS (0.8 mL) and AOS (0.8 g). After 20 min, ammonium hydroxide (24 mL; 28% ammonia solution) was added to promote the condensation reaction and hydrolysis of the silicone precursors. Sonication was continued for a further 60 min to obtain a white turbid suspension at 35 °C. 

HSNP-2 was typically prepared via an emulsion technique. In this case, a mixture of TEOS (0.8 mL), ASAS (0.4 mL), and AOS (0.4 g) were added dropwise to *n*-hexane (20 mL) over 30 min under continuous stirring. Then, ammonium hydroxide (7.0 mL; 28% ammonia solution) was added to obtain a clear solution. As the reaction proceeded at 35 °C, a homogeneous milky colloidal solution formed gradually under continuous stirring (200 rpm). The HSNP-1 and -2 particles were separated from their respective solutions via ultracentrifugation at 10000 rpm for 30 min followed by several times of washing with ethanol and drying in a vacuum oven at 35 °C. The yields (%) for HSNP-1 and HSNB-2 are 95 and 90 %, respectively. 

### 2.3. Characterization of HSNPs

The chemical structures of AOS and ASAS precursors were investigated by Fourier transform infrared (FTIR) spectroscopy (Shimadzu FTIR 8000 spectrometer, Kyoto, Japan) using KBr disc and ^1^H NMR spectroscopy (400 MHz Bruker Avance DRX-400 spectrometer, Toronto, ON, Canada) using deuterated dimethylsulfoxide (DMSO) as the solvent. The advancing and receding contact angles of water droplets on glass panels coated with hydrophobic silica capsules were measured by the sessile drop method at room temperature using a drop shape analyzer (model DSA-100, Krüss GmbH, Hamburg, Germany). The surface morphologies of the HSNPs were investigated using transmittance electron microscopy (TEM; JEOL JEM-2100F JEOL, Tokyo, Japan). Dynamic light scattering (DLS; Zetasizer Nano ZS, Malvern Instrument Ltd., Malvern, UK) was used to determine the hydrodynamic diameter (*H*_d_) and polydispersity index (PDI) of the silica nanocapsules dispersed in *n*-hexane at 25 °C. The zeta potentials were determined in methanol dispersions.

### 2.4. Surface Modification for Superhydrophobic Sand

Superhydrophobic sand was prepared by treating desert sand (5 g; 100–250 µm) with HSNP-1 or HSNP-2 (50 mL; 10 wt.% dispersed in ethanol) under continuous stirring for 3 h at room temperature. The sand was collected and dispersed in toluene and precipitated three times and dried at 60 °C for 1 h. 

Fabrication of superhydrophobic polyurethane sponge (PU) was carried out by dispersion of HSNP-1 or -2 (0.18 g), PDMS prepolymer (5 mL; Sylgard 184A), a thermal curing agent (0.05 mL; Sylgard 184B), in (60 mL) ethanol. Then, a cleaned PU sponge was immersed in the HSNP solutions for approximately 30 min, then removed and dried in an oven at 90 °C for 1 h. Here, PDMS acted as an adhesive aiding the adherence of HSNP-1 or -2 to the PU sponge.

### 2.5. Efficiency of the Superhydrophobic Sand and Hydrophobic PU Sponge in the Collection of Oil Spills

Approximately 5 mL of heavy crude oil was poured onto the surface of 250 mL of seawater in a 500 mL beaker. Different amounts of the superhydrophobic sand with respect to the crude oil content were spread as a hydrophobic powder onto the crude oil surface and remixed using a glass rod for 1 min. The remaining oil was extracted from the seawater surface using chloroform. The efficiency of the hydrophobic sand in the cleanup of the oil spill, CE (%), was evaluated using the following equation:

CE (%) = (*V*_0_/*V*_1_) × 100 (1)

where, *V*_0_ and *V*_1_ are the volumes of the removed oil and the original oil spill, respectively. The CE (%) of treated superhydrophobic PU sponge was determined by applying it at 2 cm thickness on the crude oil surface to absorb the oil without water. The reusability of the hydrophobic PU sponge was tested after pressing or squeezing the oil out of the PU sponge and washing it several times with chloroform and ethanol. The chloroform and ethanol were used to extract the oil and contaminated seawater, respectively. The chloroform was added to the swelled PU foam included crude oils with volume ratio 1:1. The remained crude oil contaminated with water was extracted with chloroform as reported in our previous works [7,8,9]. The UV-Visible spectrophotometer was used to determine the crude oil concentrations extracted with the chloroform layer by comparing the peak absorbance at 580 nm with the standard calibration curve of the crude oil.

## 3. Results

**Scheme 1:**
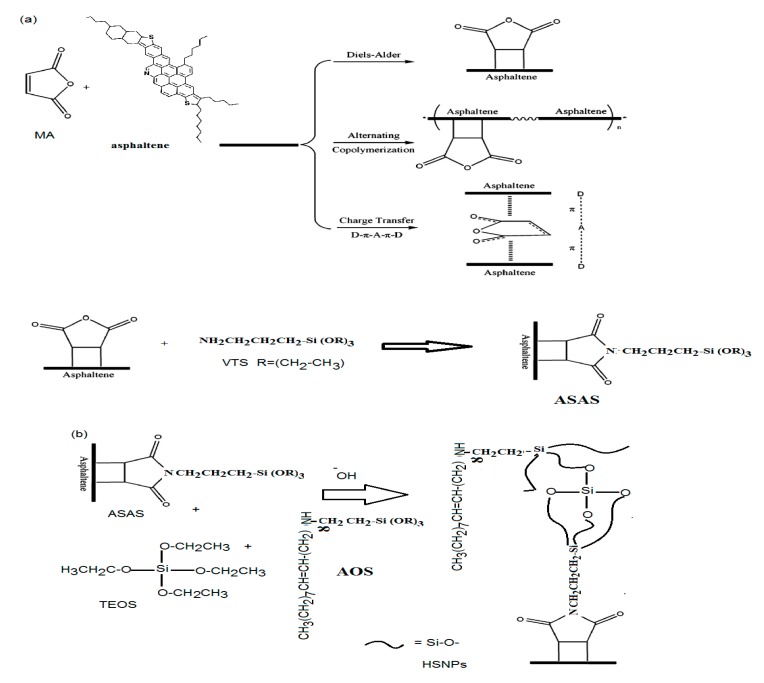
Schematic of the synthesis of (**a**) asphaltene succinimide derivative of γ-aminopropyltriethoxysilane (ASAS) and (**b**) hydrophobic silica nanoparticles (HSNPs).

In this article, we report the formation of ASAS via the formation of asphaltene-maleic anhydride adducts followed by the reaction with APS, as represented in Scheme 1a. Similarly, HSNP-2 was synthesized by the alkaline hydrolysis of silane precursors such as ASAS, TEOS, and AOS either in methanol or a hexane/water emulsion system, as illustrated in the experimental section and Scheme 1b. The asphaltenes have higher contents of fused aromatic rings, heteroatoms, metals, linear alkyl chains and higher molecular weights as compared with lighter petroleum fractions. The molecular structures and characterization of asphaltenes depend on the sources of the crude oils. The chemical structures of the asphaltenes produced from Saudi Arabian crude oils were characterized by x-ray diffraction [32]. It was reported that the asphaltenes have condensed aromatic sheets bearing naphthenic and alkyl systems on their periphery [32]. Moreover, the aromatic fractions decreased with the increasing of the API gravity of the crude oils. The reversible colloid-to solution transition structures of asphaltenes in non-polar systems were also previously reported [33]. The reaction of maleic anhydride (MA) with asphalt fractions was previously reported [34]. It has also been reported that asphaltene can react with MA either via the Diels Alder reaction mechanism or by forming jan alternating copolymer with the asphalt component [34]. The reaction of asphaltene with MA can be completed either through Diels-Alder or charge transfer mechanisms [35]. 

**Figure 1:**
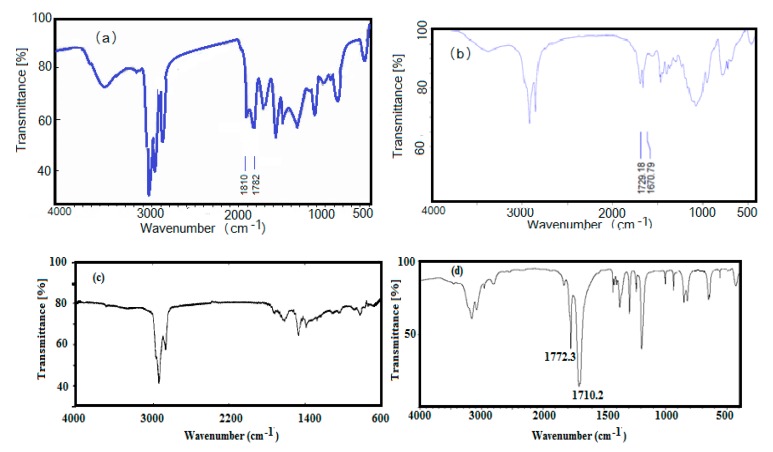
FTIR spectra of (**a**) Asphaltene-maleic anhydride, (**b**) ASAS, (**c**) asphaltene, and (**d**) succinimide.

**Figure 2:**
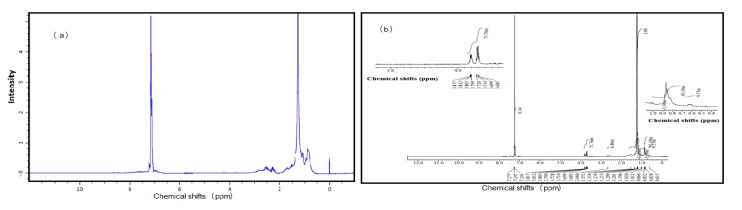
^1^H NMR spectra of (**a**) asphaltenes and (**b**) ASAS.

The proposed chemical structures of ASAS elucidated from FTIR and ^1^H NMR spectra are presented in Figure 1 and Figure 2, respectively. In the FTIR spectrum of ASAS (Figure 1b), new bands at 1670 and 1729 cm^−1^ corresponding to the C=O stretching of the succinimide group appear with the disappearance of the bands of C=O of anhydride group at 1810 and 1782 cm^−1^ observed in the spectrum of asphaltene-maleic anhydride (Figure 1a). This result confirms the reaction of the anhydride groups in asphaltene-maleic anhydride to produce succinimide, without the ring opening of the maleic anhydride group [36]. Moreover, the band at ~950 cm^−1^ is assigned to the stretching of the Si–O species. The FTIR spectra of the isolated asphaltene (Figure 1c) and succinimide (Figure 1d) are also shown for reference. The spectrum of asphaltene confirms that it contains a major portion of aliphatic –CH_2_ as inferred from the presence of strong bands at 2850 and 2920 cm^−1^ (CH_2_ stretching symmetric and asymmetric bands). However, the asphaltenes also contain a significant portion of aromatics and or C=C groups, as confirmed from the band observed at 1600 cm ^−1^ (C=C stretching). The ^1^H NMR spectra of ASAS and asphaltene (Figure 2a,b) shows new different chemical shifts at 0.95 and 3.75 ppm (broad, aliphatic protons) attributed to CH_3_ and CH_2_ groups attached to the Si–O group, which are not observed in the ^1^H NMR spectrum of asphaltene (Figure 2b). The ^1^H NMR spectrum of asphaltene (Figure 2b) indicates that it contains a significant amount of paraffinic H atoms (CH_3_, CH_2_), relatively low amounts of monoaromatic and polyaromatic H atoms (5%), and a negligible amount of olefins. Moreover, the intensity of the peaks at 1.2 ppm (S, CH_2_ aliphatic protons) and 7.26 ppm (S, aromatic protons) in both spectra (Figure 2a,b) indicate the hydrophobicity of ASAS. The increase in the intensities of the aliphatic hydrogen signals compared to those of the aromatic hydrogen signals confirms the presence of oleyl groups apart from the cyclic or linear aliphatic chains, suggesting higher hydrophobicity of ASAS compared to that of asphaltenes. The appearance of a new peak at 3.8 ppm elucidates the CH attached to the succinimide group, and it confirms the chemical structure of ASAS, as represented in Scheme 1.

### 3.1. Characterization of HSNPs

**Figure 3:**
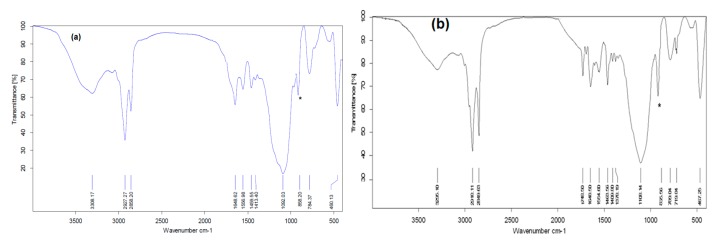
FTIR spectrum of (**a**) HSNP-1 and (**b**) HSNP-2.

The FTIR spectra of HSNP-1 and HSNP-2 are presented in Figure 3a,b, respectively. 

**Figure 4:**
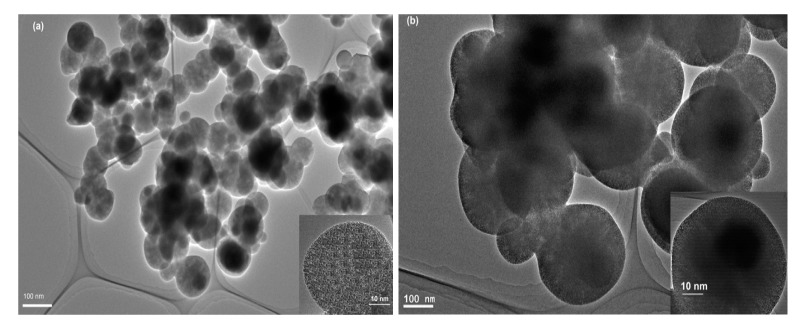
TEM micrographs (**a**) HSNPs-1 and (**b**) HSNPs-2.

The surface morphologies of HSNP-1 and -2 were investigated using TEM micrographs presented in Figure 4a,b. 

**Figure 5:**
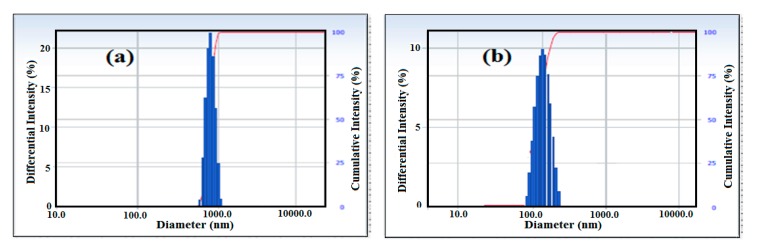
DLS data in *n*-heptane of (**a**) HSNP-1 and (**b**) HSNP-2.

**Figure 6:**
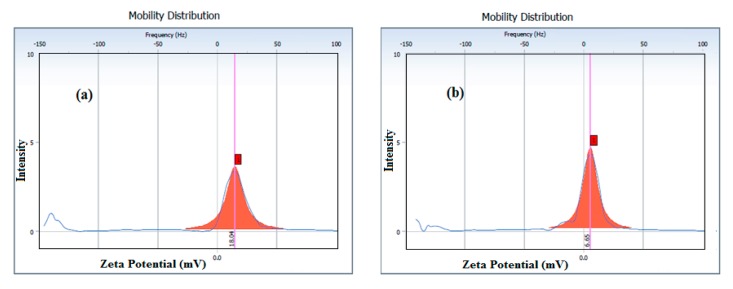
Zeta potential (mV) data of (**a**) HSNP-1 and (**b**) HSNP-2 in methanol at 25 °C.

The particle sizes and surface charges (zeta potential; mV) determined via DLS measurements are presented in Figure 5 and Figure 6a,b. 

### 3.2. Wetting Characteristics of HSNPs 

**Figure 7:**
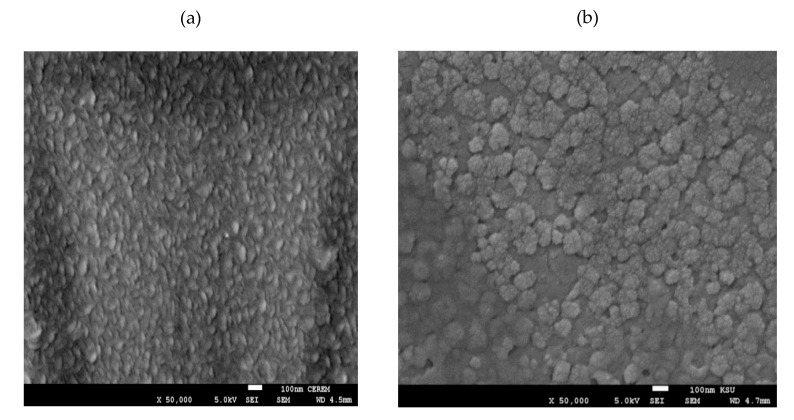
Scanning electron micrographs (SEM) micrographs (**a**) HSNP-2 and (**b**) HSNP-1.

**Figure 8:**
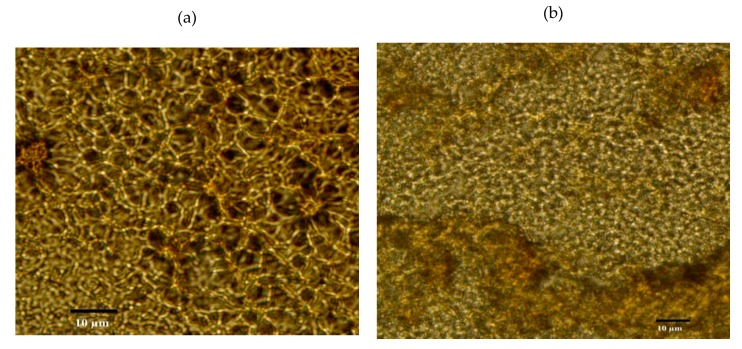
Optical micrographs of (**a**) HSNP-1 and (**b**) HSNP-2.

Scanning electron micrographs (SEM) and optical micrographs of the hydrophobic surfaces of HSNP-1 and HSNP-2 are presented in Figure 7 and Figure 8a,b, respectively. 

**Figure 9:**
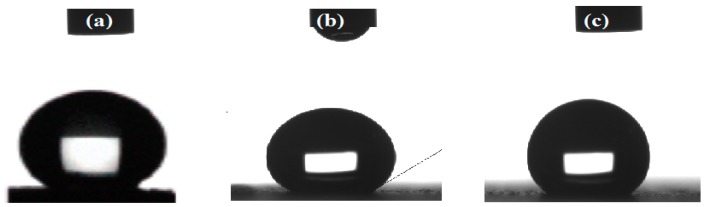
Contact angles of water on HSNP-2 surfaces (HSNP-2 samples were prepared with different contents of ASAS (**a**) 40, (**b**) 10, and (**c**) 5 wt.% at 25 °C).

The advancing contact angles of HSNP-2 modified with different wt.% of ASAS are summarized in Table 1 and represented in Figure 9. 

**Figure 10:**
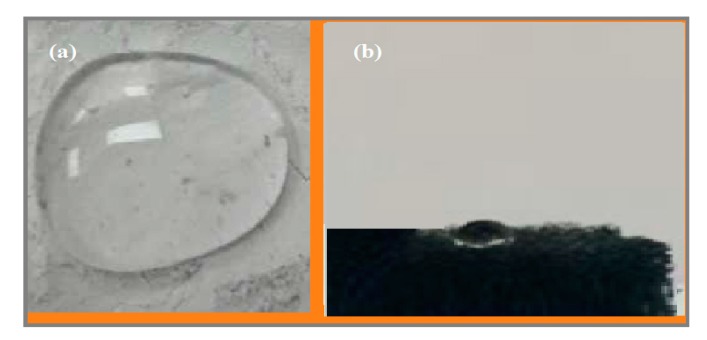
Water droplet on the surface of (**a**) hydrophobic sand and (**b**) hydrophobic polyurethane (PU) sponge modified with HSNP-2 (prepared using 40 wt.% ASAS).

The shape of water on sand and PU sponge treated with HSNP-2 (prepared using 40 wt.% ASAS) are selected and presented in Figure 10a,b. 

### 3.3. Efficiency of the Treated Superhydrophobic Sand and PU Sponge as Oil Spill Collectors

The ability of superhydrophobic HSNP-2 mixed with sand at different weight ratios ranging from 1:2 to 1:8 was studied. The treated sand was added at different weight ratios with respect to the crude oil (sand:crude oil = 1:1 to 1:10) to clean up the oil spilled on water, as reported in the experimental section. The sand mixtures were added to the surfaces of the Arabian heavy crude oil and their efficiencies, as collectors were determined as reported in the experimental section. The CE% determined three parallels and their values were listed in Table 2.

**Figure 11:**
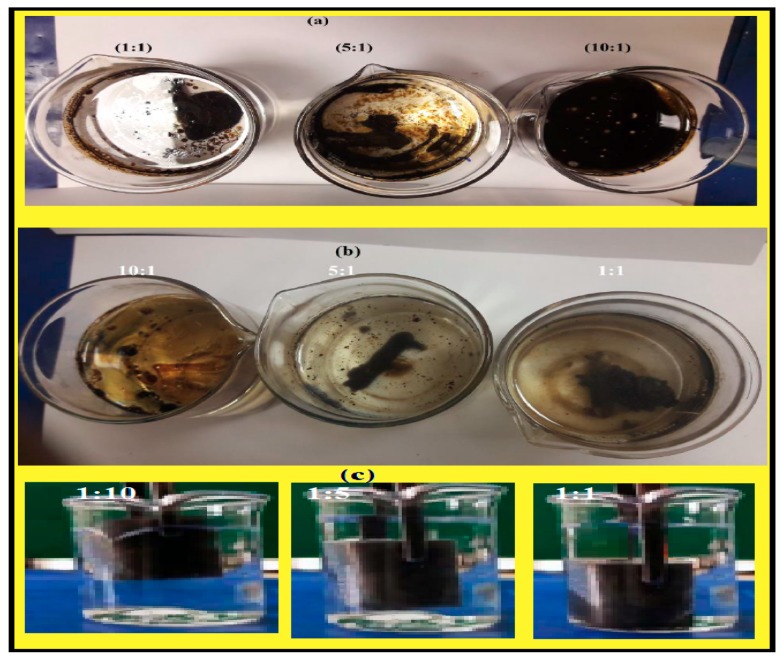
Images showing oil spill collection with (**a**) superhydrophobic sand, (**b**) HSNP-2 (prepared in the presence of 40 wt.% ASAS), and (**c**) PU sponge treated with HSNP-2 (prepared in the presence of 40 wt.% ASAS).

The PU sponge modified with HSNP-2 at 1:1 ratio (HSNP-2 was prepared in the presence of 40 wt.% ASAS) achieved 100% oil spill collection in few seconds. The corresponding photos are shown in Figure 11a–c. 

## 4. Discussion

The HSNPs can be prepared by the alkaline hydrolysis of ASAS, TEOS, and AOS (Scheme 1). It is expected that the hydrophobic interaction or π–π interaction of the aromatic groups of asphaltene and the double bond of oleyl group between ASAS and AOS increases the formation of micelles during the hydrolysis of the silane precursors. Therefore, the alkaline hydrolysis of silane precursors can be carried out in methanol or in a hexane/water emulsion system [37]. The silane precursors (ASAS and AOS) and TEOS used as the oil phase in bulk or mixed with *n*-hexane were hydrolyzed using ammonia at 35 °C to obtain HSNP-2. The FTIR spectra of HSNPs (Figure 3a,b) elucidated the increased intensity of the band at 3300 cm^−1^ (SiO–H stretching) for HSNP-1 (Figure 3a) compared to that of HSNP-2 (Figure 3b). This observation indicates that HSNP-1 has a higher content of hydroxyl group, and hence, higher hydrophilicity than HSNP-2. This means that HSNP-2 is more hydrophobic than HSNP-1. Moreover, the appearance of the band at 865 cm^−1^ in Figure 3 (Si-OH stretching band) confirms the covalent bonding of Si–OH groups produced from the hydrolysis of the ether groups of TEOS with the silicone precursors to form silica nanoparticles [38]. The disappearance of the band at 960 cm^−1^ (Si-OH stretching) also confirms that the HSNPs are formed via Si–O–Si bonding. The presence of bands in the range of 1726 cm^−1^ to 1748 cm^−1^ and that at 1726 cm^−1^ (CO–N– stretching) in the FTIR spectrum of HSNP-2 (Figure 3b) confirms the stability of succinimide during the alkaline hydrolysis of ASAS and the resultant formation of HSNPs. The shift of the imide band from 1729 cm^−1^ (Figure 1b) of ASAS to 1742 cm^-1^ (Figure 3b) elucidates the increasing intramolecular interaction among ASAS functional groups with imide groups. The FTIR spectra of HSNPs elucidate the formation of SNPs with Si–O–Si bonding without the hydrolysis of the succinimide groups bonded with asphaltenes. Furthermore, this data proves that the alkaline hydrolysis of silane precursors using the emulsion technique in hexane/water increases the hydrophobic interactions between the hydrophobic groups or π–π interaction of the polar groups of the reactants. The TEM micrographs (Figure 4) reveal the formation of spherical HSNPs. The high-resolution TEM images of the nanoparticles are also shown in the bottom panel of Figure 4. Uniform spherical core/shell morphology is observed for HSNP-2 as compared to that of HSNP-1. Moreover, aggregation of HSNP-1 is observed (Figure 4a). The core/shell morphology of HSNP-2 was referred to the strong interaction between the hydrophobic groups of ASAS, and AOS tends to facilitate the assembly of the hydrophobic groups in hexane and hydrophilic groups in water during the formation of HSNP-2. The uniform spherical morphology of HSNPs-2 may be attributed to the surface charges of the nanoparticles. It is recommended to measure the particle sizes of the functionalized materials by DLS than TEM techniques. The TEM measurements evaluate the particle sizes in the dry state. The DLS technique is preferred to evaluate the particle sizes which controlled either by the interactions of particles with the solvent or their surface charges. In this respect, the DLS measurements of HSNPs-1 and HSNPs-2 as superhydrophobic particles (Figure 5a,b) were measured in hydrophobic solvent such as n-hexane. The particle size (nm) and PDI of HSNP-1 are 958.16 nm and 0.251, respectively, whereas the particle size and PDI of HSNP-2 are 105.5 nm and 0.156, respectively. These data elucidate that HSNP-2 has lower PDI and lower particle size than HSNP-1. This result can be due to the increased hydrolysis of the alkoxy groups of TEOS and ASAS in the emulsion system. Accordingly, the good compatibility between the reactants reduces the particle sizes of the nanoparticles [39]. The surface of HSNP-2 (Figure 6a) is less positively charged compared to that of HSNP-1, to confirm that the absence of ASAS during the synthesis of HSNPs enhances the appearance of hydroxyl groups on the surface of silica. The presence of ASAS during the preparation of HSNP-2 changes the surface charge of the particles to positive; this results from imide groups of ASAS and asphaltene charges in polar and nonpolar solvents [40]. 

Changing the hydrophilic surfaces of the PU sponge and sand to superhydrophobic surfaces is proposed for selectively adsorbing crude oil from water surfaces. Increasing the wetting characteristics of sand or PU sponge for organic phases is an active challenge in materials science and is a requirement for them to be applicable in the petroleum industry. It has been previously reported that superhydrophobic sand with different degrees of wettability can be used in the separation of emulsions or treatment of contaminated water [41]. The fabrication of superhydrophobic porous materials based on the PU sponge has great potential for oil absorption owing to their good stability, high absorption capacity, and recyclability [42]. The SEM micrographs (Figure 7 and Figure 8) indicate that HSNP-2 films (Figure 7a and Figure 8b) have a more uniform rough surface than those of HSNP-1 (Figure 7b and Figure 8a). This may be attributed to the good ability of HSNP-2 to form monodisperse nanoscale surfaces than that of HSNP-1, which forms aggregates [43]. The fragility of the nanoscale roughness features of HSNP-1 and HSNP-2 films was evaluated by measuring the contact angles of water droplet on their surfaces, to determine their wettability. Unfortunately, the contact angle of water on HSNP-1 surface could not be evaluated, owing to the formation of a non-uniform film on the glass surface. Therefore, the HSNP-1 particles were adhered to the glass plate using adhesive tape (3M) to obtain a film with a thickness of 100 µm to measure the contact angle. The water contact angle on the surface of this HSNP-1 film is 101.23 ± 3°. The wetting of the HSNP-2 particles was evaluated by applying a thin film of the particles at a thickness of 50 μm on glass panels. Different HSNP-2 particles were prepared by varying the amount of ASAS from 40 to 5 wt.% and applied on glass surfaces to determine the contact angles. The water contact angle of the untreated glass surface is 45°. The contact angle data (Table 1 and Figure 9) indicate a higher contact angle for HSNP-2 prepared in the presence of 40 wt.% ASAS, with a value of 170°. All the HSPN-2 particles modified with ASAS formed superhydrophobic surfaces (contact angle > 150°), except the one modified with 5 wt.% ASAS. The data presented in Figure 10a,b confirm that HSNP-2 forms superhydrophobic rough films on sand and PU sponge surfaces. The spherical morphology of HSNP-2 (Figure 4b) is responsible for the formation of rough surfaces, apart from its bonding adhesion with the silicate bond of sand and its bonding deposition in PU pores [42].

The data of oil spill collection efficiency, summarized in Table 2, indicates that the efficiency of the hydrophobic sand capped with HSNP-2 in the collection of oil spills from water surfaces increases with an increase in the sand:crude oil ratio. The sand:crude oil of 1:1 was the best for achieving a CE % of 92%. It was also observed that the crude oil adhered on the treated superhydrophobic sand surfaces could be removed by immersion in toluene. The good crude oil collection with the superhydrophobic sand treated with HSNP-2 can be due to the good dispersion of superhydrophobic sand in crude oil, which leads to a change in the surface properties of both water and oil at the interface. The dispersion of superhydrophobic sand in the crude oil assists in repelling the crude oil from the water surface and facilitates the crude oil collection [44]. It is observed from Figure 11a–c, that the treated PU sponge selectively absorbed crude oil without any water. Moreover, it is also observed that the PU sponge can separate large amounts of crude oil, reaching 50 times its original weight. Compared to the previously reported inorganic graphene aerogel and poly(dimethylsiloxane) sponge [12,45], the PU sponge treated with HSNP-2 has an excellent absorption capability with CE of 95.6 % for heavy crude oil during a 2 min period. This observation suggests that the PU sponge treated with HSNP-2 has good selectivity for the rapid cleanup of the crude oil spill without water. In addition, the adsorbed crude oil can be recycled by squeezing out the oil from the PU sponge treated with HSNP-2 under simple compression. The PU sponge treated with HSNP-2 recovered its original form and could be reused to collect crude oil up to five times without reduction in its CE %. The small decrease in the CE % of the PU sponge treated with HSNP-2 after five cycles can be attributed to the entrapment of the crude oil in the pores of the sponge, which prevent it from absorbing crude oil or water.

## 5. Conclusions

Novel superhydrophobic silica nanoparticles were prepared in the presence and absence of ASAS silane precursors to obtain monodisperse uniform spherical nanoparticles via an emulsion technique. The HSNP-2 samples prepared in the presence of different amounts of ASAS precursor formed rough and superhydrophobic films. They were used as coatings to obtain superhydrophobic sand and PU sponge. The good crude oil collection of the superhydrophobic sand treated with HSNP-2 can be attributed to the good dispersion of superhydrophobic sand in the crude oil. Sand modified with the HSNPs dispersed in the petroleum crude oil to increase the specific gravity of the crude oil, leading to the coalescence of the crude oil from the water surface. The hydrophobic sand could not be easily separated from crude oil in water owing to its good dispersion in the crude oil and its superhydrophobicity. The PU sponge treated with HSNP-2 facilitated fast removal of heavy crude oil from water surface. Furthermore, its original form could be recovered, and it could be reused to collect crude oil up to five times without reduction in its CE. Therefore, it has great potential to be applied as an adsorbent in the large-scale removal of petroleum crude oil spills and hydrophobic organic pollutants in marine and aquatic systems. 

## Figures and Tables

**Table 1:** Advancing contact angles of water droplets on HSNP-2 surfaces at 25 °C.

HSNP-2 (ASAS wt.%)	Contact Angle (degree)
40	170 ± 4
20	160 ± 3
10	155 ± 4
5	140 ± 2

**Table 2:** Efficiency of the hydrophobic sand capped with HSNP-2 (prepared using 40 wt.% ASAS) for the collection of the oil spills from water surfaces.

Ratio of HSNP-2: Sand (Wt.:Wt.)	Ratio of Sand:Crude oil (Wt.:Wt.)	Efficiency (CE%)
(1:2)	1:1	92 ± 1
	1:2	90 ± 2
	1:10	80 ± 3
(1:4)	1:1	89 ± 3
	1:2	85 ± 4
	1:10	58 ± 5
(1:8)	1:1	78 ± 3
	1:2	55 ± 4
	1:10	28 ± 5
